# The impact of phosphodiesterase inhibition on neurobehavioral outcomes in preclinical models of traumatic and non-traumatic spinal cord injury: a systematic review

**DOI:** 10.3389/fmed.2023.1237219

**Published:** 2023-08-22

**Authors:** Max B. Butler, Sundar K. Vellaiyappan, Faheem Bhatti, Fazal-E-Momin Syed, Amir Rafati Fard, Jye Quan Teh, Ben Grodzinski, Melika Akhbari, Sylva Adeeko, Rory Dilworth, Aniqah Bhatti, Unaiza Waheed, Sophie Robinson, Temidayo Osunronbi, Benn Walker, Luke Ottewell, Gayathri Suresh, Isla Kuhn, Benjamin M. Davies, Mark R. N. Kotter, Oliver D. Mowforth

**Affiliations:** ^1^Division of Academic Neurosurgery, Department of Clinical Neurosciences, University of Cambridge, Cambridge, United Kingdom; ^2^Medical Library, University of Cambridge, Cambridge, United Kingdom

**Keywords:** spinal cord, spinal cord injury, phosphodiesterase inhibitor, preclinical model, neurobehavioral outcomes

## Abstract

**Study design:**

Systematic review.

**Objective:**

The objective of this study was to evaluate the impact of phosphodiesterase (PDE) inhibitors on neurobehavioral outcomes in preclinical models of traumatic and non-traumatic spinal cord injury (SCI).

**Methods:**

A systematic review was conducted following the Preferred Reporting Items for Systematic Reviews and Meta-Analysis (PRISMA) guidelines and was registered with PROSPERO (CRD42019150639). Searches were performed in MEDLINE and Embase. Studies were included if they evaluated the impact of PDE inhibitors on neurobehavioral outcomes in preclinical models of traumatic or non-traumatic SCI. Data were extracted from relevant studies, including sample characteristics, injury model, and neurobehavioral assessment and outcomes. Risk of bias was assessed using the SYRCLE checklist.

**Results:**

The search yielded a total of 1,679 studies, of which 22 met inclusion criteria. Sample sizes ranged from 11 to 144 animals. PDE inhibitors used include rolipram (*n* = 16), cilostazol (*n* = 4), roflumilast (*n* = 1), and PDE4-I (*n* = 1). The injury models used were traumatic SCI (*n* = 18), spinal cord ischemia (*n* = 3), and degenerative cervical myelopathy (*n* = 1). The most commonly assessed outcome measures were Basso, Beattie, Bresnahan (BBB) locomotor score (*n* = 13), and grid walking (*n* = 7). Of the 22 papers that met the final inclusion criteria, 12 showed a significant improvement in neurobehavioral outcomes following the use of PDE inhibitors, four papers had mixed findings and six found PDE inhibitors to be ineffective in improving neurobehavioral recovery following an SCI. Notably, these findings were broadly consistent across different PDE inhibitors and spinal cord injury models.

**Conclusion:**

In preclinical models of traumatic and non-traumatic SCI, the administration of PDE inhibitors appeared to be associated with statistically significant improvements in neurobehavioral outcomes in a majority of included studies. However, the evidence was inconsistent with a high risk of bias. This review provides a foundation to aid the interpretation of subsequent clinical trials of PDE inhibitors in spinal cord injury.

**Systematic review registration:**

https://www.crd.york.ac.uk/prospero/display_record.php?RecordID=150639, identifier: CRD42019150639.

## Introduction

Spinal cord injury (SCI) has a prevalence that ranges from 250 cases per million in the Rhone-Alpes region of France to 906 cases per million in the United States of America (1). It encompasses sensory, motor, and autonomic impairments with severe consequences for physical, psychological, and social health (2).

The initial phase of SCI arises directly from mechanical trauma on the spinal cord. This triggers a secondary phase of damage from mechanisms including excitotoxicity, ischemia, and inflammation (3, 4). Neutrophils and macrophages release cytokines, proteolytic enzymes, and reactive oxygen species, resulting in damage to neurons, glia, and vascular structures (5). Vascular impairment may catalyze increased activation of voltage-gated sodium channels, leading to sodium influx and cell swelling (6, 7). Disrupted calcium homeostasis triggers glutamate release, leading to neuronal excitotoxicity and cell death (8, 9). Drugs with anti-inflammatory effects, such as phosphodiesterase (PDE) inhibitors, may therefore have efficacy in reducing the irreversible spinal cord damage that ensues from these secondary mechanisms of injury (10).

PDEs are enzymes that have proinflammatory effects, in part through degrading cyclic adenosine monophosphate (cAMP), which regulates microglia homeostasis and inflammatory cytokine expression (11). By elevating cAMP levels, PDE inhibitors have been shown to reduce inflammatory cytokine production (9, 12, 13) and promote central nervous system (CNS) regeneration (14). PDE4 is the most frequently expressed cAMP-specific PDE in neurological tissue (15) and monocytes (16) and is a therapeutic target in inflammatory disease (17).

Given the pathophysiology of SCI, adjuvant therapy with PDE inhibitors may prove beneficial through mechanisms including remyelination (18). For example, rolipram is a non-specific PDE4 inhibitor that has diverse anti-inflammatory properties (19–22) and inhibitory effects on glutamate toxicity, caspases (23, 24), and neurite outgrowth inhibition (25). Rolipram has been used both in experimental models and as a clinical therapy for asthma (26), arthritis (27), Huntington's disease (28), multiple sclerosis (21), Alzheimer's disease (29), human immunodeficiency virus (HIV) (30), and traumatic brain injury (31, 32). In addition, cilostazol, a selective PDE3 inhibitor, has been shown to have neuroprotective effects in ischemic cerebral injury (33–35) and diabetic retinal dysfunction (36). PDE3 inhibitors have also been found to have antiplatelet (36), antithrombotic (37), and vasodilatory effects (38). The role of PDE inhibition is currently being evaluated in RECEDE Myelopathy, a phase III randomized trial of the PDE4 inhibitor ibudilast in patients with degenerative cervical myelopathy (39).

The aim of this systematic review was to evaluate the impact of PDE inhibitors on neurobehavioral outcomes in preclinical models of traumatic and non-traumatic SCI.

## Methods

This systematic review was conducted following the Preferred Reporting Items for Systematic Reviews and Meta-Analysis (PRISMA) guidelines (40).

### Protocol and registration

The protocol was published on PROSPERO (CRD42019150639).

### Eligibility criteria

The inclusion and exclusion criteria used in this review are presented in Table 1.

**Table 1:** Inclusion and exclusion criteria.

	**Inclusion**	**Exclusion**
Population	Any animal model, including: • Rats • Mice • Rabbits	• Humans
Injury models	• Traumatic spinal cord injury • Degenerative cervical myelopathy • Spinal cord ischemia	• Non-spinal pathologies • Root evulsion injuries • Peripheral nerve injuries (e.g., sciatic nerve) • Traumatic brain injury • Epilepsy • Parkinson's disease • Amyotrophic lateral sclerosis • Transverse myelitis
Intervention	PDE inhibitor delivered: • Intravenously • Intraperitoneally • Intrathecally • in implanted drug-eluting materials	N/A
Comparison	Control (vehicle injection)	• Non-drug treatments • Other drug preparations
Outcomes	Neurobehavioral assessment, such as: • BBB locomotor score • BMS locomotor score • Grip strength • Gait analysis • Footprint analysis • Grid walk • Mechanical or thermal allodynia	• Autonomic function or physiological parameters, for example, ° Respiratory function ° Heart rate ° Temperature • *In vitro* assessments

### Population and injury model

The focus was animal models, including those using rats, mice, or rabbits. Studies involving humans were excluded. SCI models such as traumatic injury, degenerative cervical myelopathy (DCM), or spinal cord ischemia were included; injury models such as peripheral nerve injury or traumatic brain injury were excluded.

### Intervention and comparison

Studies were included if they utilized PDE inhibitors, such as rolipram and cilostazol, administered intravenously, intraperitoneally, intrathecally, or via implanted drug-eluting materials. To be included, studies required a control treatment group and at least one PDE inhibitor treatment group. Studies were not excluded based on drug administration parameters such as size, frequency, or duration of dosing.

### Outcomes

Neurobehavioral outcomes were the focus of this review. Studies that involved any neurobehavioral outcome, such as Basso, Beattie, and Bresnahan locomotor score, grid walking assessment, and mechanical or thermal allodynia were included. Studies that only assessed parameters such as histological or autonomic outcomes were excluded.

### Information sources

A systematic search was performed of MEDLINE and Embase databases from inception until 10 January 2023.

### Search

The search strategy was developed with the assistance of a medical librarian (IK) at the University of Cambridge Medical Library. The terms used to search MEDLINE and Embase are provided in Supplementary material 1. No additional search limits were applied.

### Study selection

Duplicates were excluded in EndNote (Clarivate, London, UK). The abstracts were then screened independently by 19 authors using Rayyan software. Following an initial pilot of 100 articles, reviewers met to resolve disagreements and ensure consistency in the interpretation of inclusion criteria. Abstracts were then divided into seven groups. Each group was screened in duplicate by a pair of reviewers. Disagreements were resolved through discussion between the reviewers.

### Data extraction

The data extracted were author, year of publication, country of experiments, study characteristics (e.g., number of experimental groups and level of evidence), sample characteristics (e.g., size, number of groups, animal species, age, sex, weight, and comorbidities), intervention (including injury model and the type, dose, frequency, and route of drug), the methods and results of any neurobehavioral assessment, and the nature of any relevant statistical analysis performed. Data were extracted by one reviewer (MB).

### Data synthesis

Due to heterogeneity in injury models, interventions, and outcome reporting, a narrative synthesis was conducted using the Synthesis Without Meta-analysis (SWiM) guideline (41).

### Risk of bias in individual studies

The SYRCLE (Systematic Review Center for Laboratory Animal Experimentation) tool was used to evaluate the risk of bias in included studies. The checklist is a modification of the Cochrane Collaboration risk-of-bias tool (42) using only the components that are directly applicable to animal selection (Table 2) (43). This checklist includes 10 domains relating to 6 forms of bias: selection, performance, detection, attrition, reporting, and other biases.

**Table 2:** Systematic review center for laboratory animal experimentation (SYRCLE) tool (43).

**Question**	**Type of bias**
Was the allocation sequence adequately generated and applied?	Selection bias
Were the groups similar at baseline or were they adjusted for confounders in the analysis?	Selection bias
Was the allocation adequately concealed?	Selection bias
Were the animals randomly housed during the experiment?	Performance bias
Were the caregivers and/or investigators blinded from knowledge of which intervention each animal received during the experiment?	Performance bias
Were animals selected at random for outcome assessment?	Detection bias
Was the outcome assessor blinded?	Detection bias
Were incomplete outcome data adequately addressed?	Attrition bias
Are reports of the study free of selective outcome reporting?	Reporting bias
Was the study apparently free of other problems that could result in high risk of bias?	Other

## Results

### Study selection

**Figure 1:**
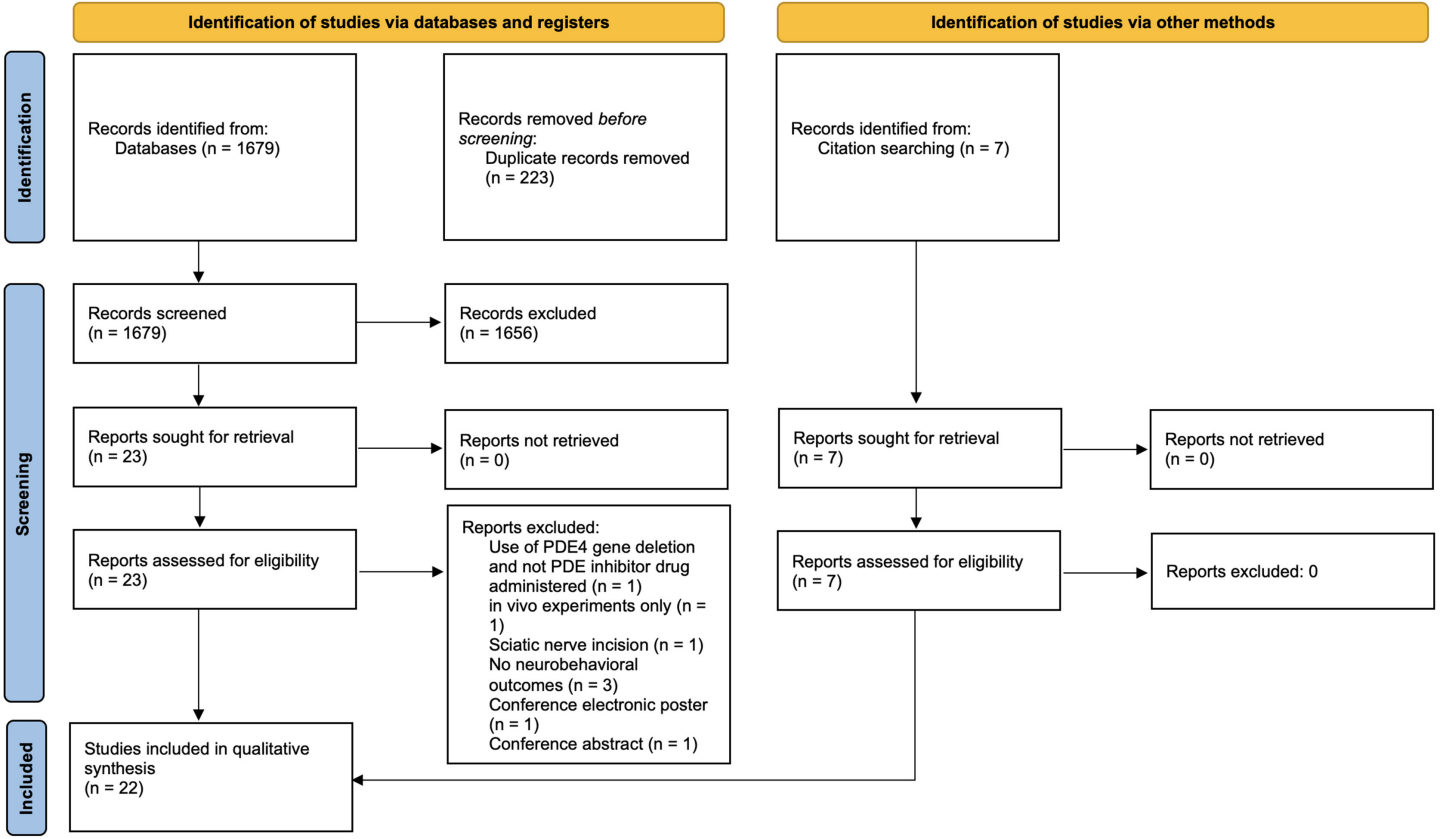
PRISMA flow diagram of study selection.

The search generated 1,679 results. A total of 223 duplicates were removed using EndNote, resulting in 1,456 unique studies, of which 23 were found to satisfy inclusion criteria following title and abstract screening. During full-text screening, eight studies were excluded for the reasons outlined in Supplementary material 2. In total, seven additional relevant studies were found on reviewing the reference lists of included studies. In total, 22 studies were therefore included in the review (Figure 1).

### Study characteristics

**Figure 2:**
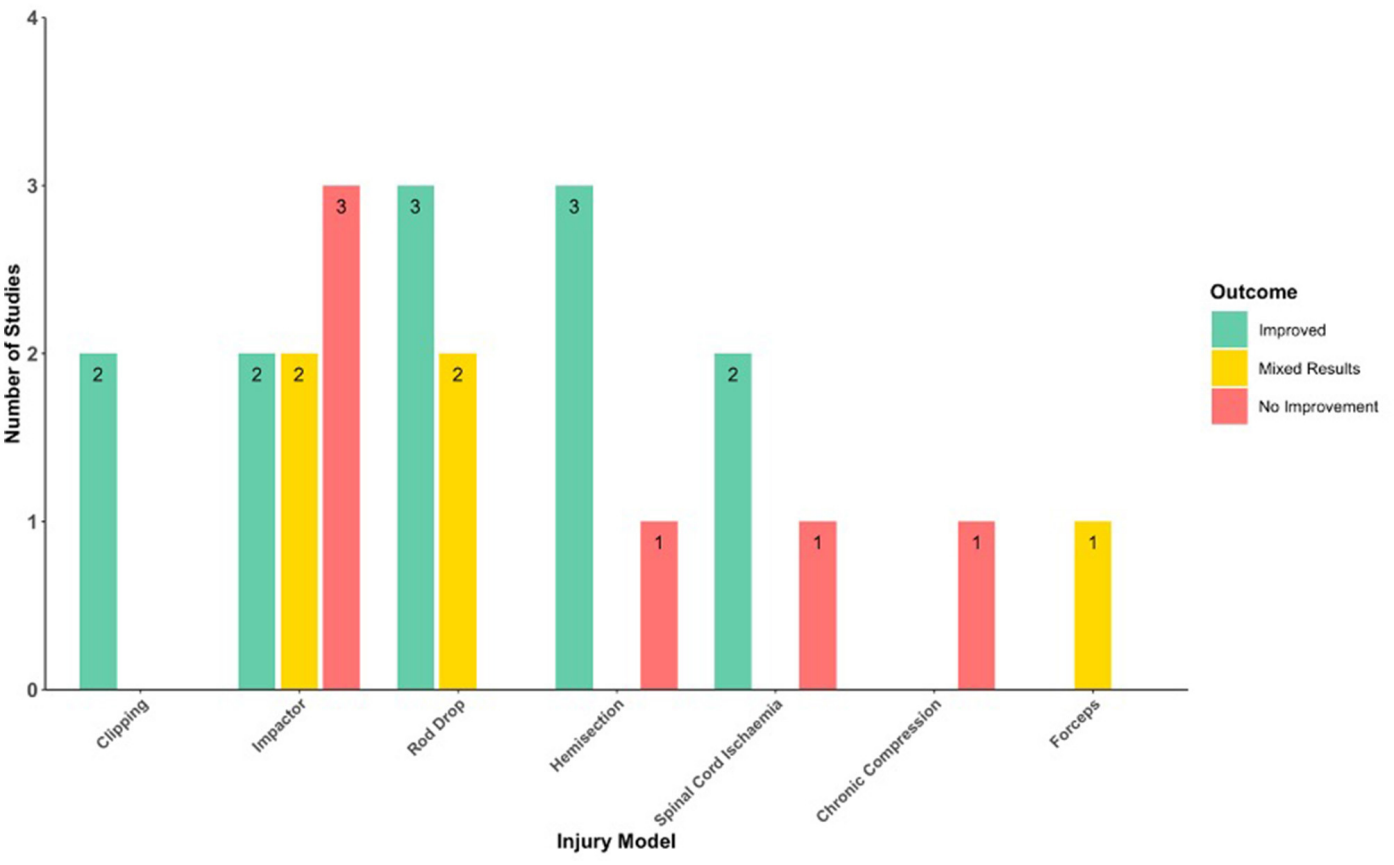
Histogram of the neurobehavioral outcomes following PDE inhibition in different injury models.

**Figure 3:**
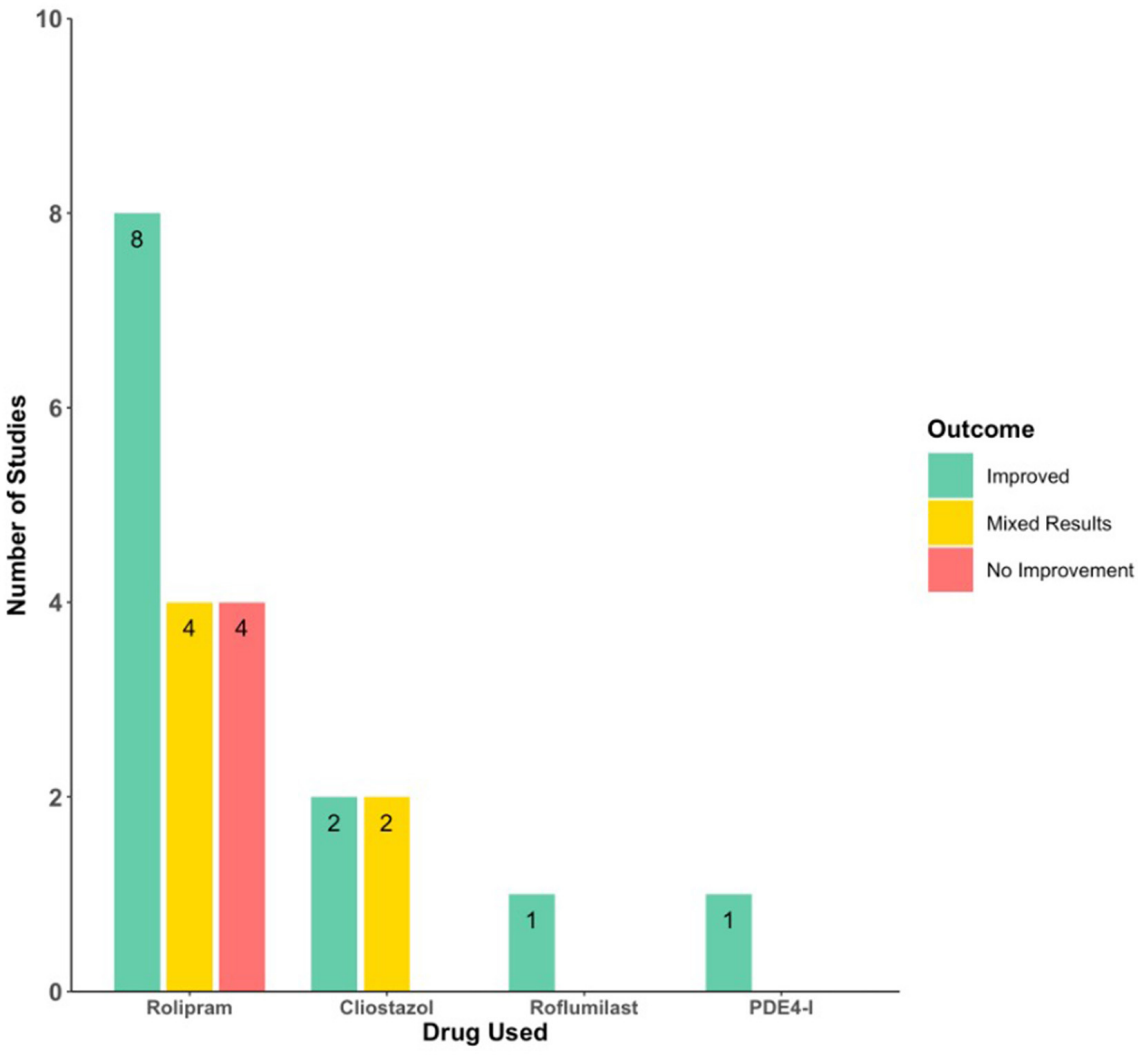
Histogram of the neurobehavioral outcomes following different PDE inhibitors.

Studies utilized either rat, mouse, or rabbit models of spinal cord injury. Models included acute SCI via spinal cord impaction devices (44–49), rod dropping (31, 50–52), microscissors (53, 54), microvascular clips (55, 56), scalpel blade incision (57), crushing with forceps (15); spinal cord ischemia via aortic clamping (58–60); and DCM via an expanding polymer insert (61) (Figure 2). Acute SCI models were either at the thoracic (*n* = 12) (31, 45–49, 51–53, 55, 56, 62) or cervical (*n* = 5) (44, 54, 57, 63, 64) level. The PDE inhibitors used were rolipram (*n* = 16) (31, 44–54, 57, 62–64), cilostazol (*n* = 4) (58–61), roflumilast (*n* = 1) (56), and PDE4-I (*n* = 1) (55) (Figure 3; Table 3). The most commonly assessed outcome measures (Table 4) were BBB (Basso, Beattie, and Bresnahan) locomotor score (*n* = 13) (31, 44–52, 55, 56, 62) and grid walking (*n* = 7) (44, 46, 50, 52, 53, 62, 63). Table 5 summarizes the sample features, injury models, interventions, outcomes, and assessments of the included studies.

**Table 3:** Summary of drugs used in experiments.

**Drug name**	**Mechanism of action**
Rolipram	Selective inhibitor of PDE4. Rolipram binds to the same site as AMP in phosphodiesterase 4B, occupying most of the binding site except for an empty pocket near its pyrrolidinone group (65). PDE4 is mainly found in immune cells, epithelial cells, and brain cells (17).
Clodronate	A bisphosphonate drug which is taken up by osteoclasts and inhibits farnesyl pyrophosphate synthase enzyme. Clodronate is intracellularly metabolized to an analog of ATP that is cytotoxic to macrophages *in vitro* by causing a collapse of the mitochondrial membrane potential (66).
Roflumilast	Long-acting selective PDE4 A-D subtype inhibitor with sub-nanomolar potencies (67) commonly used in treating patients with COPD and asthma.
Chondroitinase ABC (ChABC)	Derived from *Proteus vulgaris*, ChABC degrades the glycosaminoglycan side chains of chondroitin sulfate (CS) (68). CS chains are known to inhibit neuronal regeneration and their degradation improves nerve plasticity (69).
Cilostazol	PDE3 inhibitor approved by FDA for use in patients with intermittent claudication associated with end-stage peripheral vascular disease (70). PDE3A is found mainly in cardiac muscles, smooth muscles, and platelets while PDE3B is found in hepatocytes, adipocytes, and pancreatic cells (71).
Methylprednisolone	Anti-inflammatory drug that binds to and activates glucocorticoid receptors. Similar to other corticosteroids, methylprednisolone inhibits cyclooxygenase-2 (COX-2) synthesis as well as leukocyte and T-cell function (72).
Dibutyryl cAMP (db-cAMP)	A synthetic analog of cAMP that activates the PKA-mediated cAMP signaling pathway.
Thalidomide	Anti-angiogenic through inhibition of VEGF production and anti-inflammatory via inhibition of TNF-alpha production (73).
Nogo-66 receptor protein	Nogo-66 receptor proteins sequester Nogo-A, myelin-associated glycoprotein (MAG), and oligodendrocyte myelin glycoprotein (OMgp), preventing their interaction with endogenous Nogo-66 receptor (NgR) protein on axons, which have been shown to collapse axonal growth cones and inhibit spinal cord recovery after trauma (47).

**Table 4:** Summary of outcome assessment tools.

**Scales**	**Summary of tool**
Basso, Beattie, Bresnahan Locomotor score (31, 44–52, 55, 56, 62)	Assesses hindlimb movement, paw placement, weight bearing, trunk stability, tail position, and limb coordination. Scored from 0 to 21; 0 is no hindlimb movement and 21 is normal function.
Basso, Beattie, Bresnahan Locomotor subscore (31, 46, 48, 50, 51, 62)	Assesses toe clearance, paw position, trunk stability, and tail position. Scored from 0 to 7; 7 is normal function.
Modified Tarlov score (58–60)	0 = paraplegic with no movement; 1 = poor lower extremity motor function; 2 = some lower extremity motor function with good antigravity strength; 3 = sits/stands alone; 4 = weak hop/walk; 5 = normal motor function.
Grid walking (44, 46, 50, 52, 53, 62, 63)	Assesses sensory–motor coordination of limbs through recording footfall errors as a proportion of total steps (animals walk on an elevated grid). Performed with a regular (44, 53, 62) or irregular grid (46, 50, 52, 63).
Footprint analysis (46, 50, 62, 63)	Assessment of foot rotation (46, 62), base of support (46, 50, 62, 63), or stride length (46, 50, 62, 63), using video-based kinematic analysis (62, 63), paint (62), or ink (50), or an unspecified technique (47).
CatWalk gait analysis (63), CatWalk regularity index (48), and CatWalk-based BBB score (48)	Automated measurement of forelimb and hindlimb base of support, stride length, stand time, and swing time. The CatWalk regularity index (RI) is a measure of coordination defined as: RI = NSSP-4/PP^*^100 (NSSP, the number of normal step sequence patterns; PP, total number of paw placements). Traversing a walkway with a RI of 100% is considered coordinated. The CatWalk-based BBB score integrates this into the standard BBB score.
3D hindlimb kinematics (45)	Kinematic plots and joint angles extracted from 3D video recordings, using markers on five sites on the left hind limb and one on the right hind limb.
Vertical exploration/rearing (54, 63, 64)	When placed in a cylinder, animals spontaneously rear onto their hindlimbs to vertically explore the walls. Studies assessed the rates of using left and/or right forelimbs to contact the wall of a cylinder when rearing (54, 63, 64) the side of the forelimb used to contact the wall (dorsal vs. palmar) (50, 59), rates of forelimb raising above shoulder height (50), and the number of rears that occurred (50, 59).
Food-pellet reaching tasks (59, 60)	Assesses the ability to reach food through a small opening (scored from 0 to 10) (60) or off vertical shelves (59).
Beam walk (45)	Assesses the narrowest horizontal beam that can be crossed without foot slips. 1 = 7.7 cm, 2 = 6.7 cm, 3 = 5.7 cm, 4 = 4.7 cm, 3 = 3.7 cm, 2 = 2.7 cm, 1 = 1.7 cm.
Ladder beam task (62)	Assesses the number of footfall errors when crossing a ladder beam.
Voluntary exercise (61)	Rotations of the cage wheel recorded using an odometer. Expressed as a percentage of the pre-intervention injury average.
Cage activity assessment (49)	Movement inside the cage recorded using telemetry. Movement of transducer over detection fields recorded as counts per minute.
Mechanical allodynia (55)	Assess sensitivity to monofilament stimulation on plantar forepaw. Mean number of avoidance responses with 10 stimulations recorded.
Thermal allodynia (64)	Assess sensitivity to thermal stimulation on plantar forepaw. Time from stimulus onset to withdrawal was recorded for both forepaws. The latency of withdrawal was recorded for both forepaws.
Forced locomotion capability (61)	A measurement of the duration of time spent running on a treadmill before falling.
Grip strength (61)	Animals grip a bar, which is pulled away until it is released. The maximum force applied to the bar is recorded.
Martinez forelimb open-field score (57)	Assesses articular movement of the shoulder, elbow, and wrist; stationary and active weight support, digit position, stepping, forelimb–hindlimb coordination, and tail position. Scored from 0 to 20; 20 is a normal function.

**Table 5:** Summary of included studies.

**References, location**	**Sample features**	**Injury model**	**Intervention**	**Outcomes assessed**	**Time of assessment**
Bao et al. (55), Canada	*n* = 23 Male Wistar rats	Traumatic SCI Microvascular clip (35 g) clamped around the spinal cord for 1 min Level: T4	• PDE4-I 0.5 mg/kg (*n* = 6) • PDE4-I 1 mg/kg (*n* = 6) • Vehicle **PDE4-I** *(selective phosphodiesterase type 4 inhibitor):* 0.5 or 1 mg/kg delivered i.v. at 2, 12, 24, 36, 48, 60 h post-SCI **Vehicle:** 30% DMSO (dimethyl sulfoxide) delivered i.v. at 2, 12, 24, 36, 48, 60 h post-SCI	• BBB locomotor score • Mechanical allodynia	• BBB: pre-SCI; post-SCI from 3 days to 8 weeks (twice per week) • Mechanical allodynia: pre-SCI; post-SCI weekly at 2–6 weeks
Beaumont et al. (44), USA	*n* = 11 (+ 1 removed before analysis) Adult female Sprague–Dawley rats	Traumatic SCI Contusive injury using an impactor (175-kdyn) Level: C5-6	• Rolipram (*n* = 5 + 1 removed) • Vehicle (*n* = 6) **Rolipram:** 0.5 mg/kg/day delivered s.c. by mini-osmotic pump for 0–14 days post-SCI **Vehicle:** DMSO delivered s.c. by mini-osmotic pump for 0–14 days post-SCI	• BBB locomotor score • Grid walking	• 5 weeks post-SCI
Costa et al. (45), Portugal	*n* = 34 Adult female Wistar rats Weight: 200 g	Traumatic SCI Contusive injury using impactor (200-kdyn) Level: T10	• Rolipram (*n* = 15) • Vehicle (*n* = 15) • Sham-operated (*n* = 4) **Rolipram:** 3.18 mg/kg/day delivered s.c. by mini-osmotic pump for 0–14 days post-SCI **Vehicle:** DMSO delivered s.c. by mini-osmotic pump for 0–14 days post-SCI	• BBB locomotor score • Beam walk • 3D hindlimb kinematics	• BBB: pre-SCI; post-SCI at 2 days then weekly at 8 weeks. • Beam walk: pre-SCI; post-SCI weekly at 2–8 weeks. • 3D hindlimb kinematics: 8 weeks post-SCI
Flora et al. (50), USA	*n* = 90 Adult female Fischer rats Weight: 180–200 g	Traumatic SCI Contusive injury by 10-g rod drop from height (25 mm) Level: T9	• GFP-transduced SC + rolipram (*n* = 18) • GFP-transduced stem cells (SC; *n* = 22) • GFP-D15A-cotransduced SC + rolipram (*n* = 16) • GFP-D15A-cotransduced SC (*n* = 16) • Vehicle (*n* = 18) **Rolipram:** 1.0 μl/h of 0.5 mg/kg delivered s.c. by mini-osmotic pump for 0–28 days post-SCI **Stem cells:** 2 million cells injected into the injury center in a 1:1 mix of SCs/DMEM/F12 and Matrigel, transduced with an enhanced green fluorescent protein (GFP) and/or a bifunctional neurotrophin molecule (D15A) 1 week post-SCI) **Vehicle:** DMEM/F12/Matrigel without cells, 8 ml of total volume injected into lesion center 1 week post-SCI	• BBB locomotor score • BBB locomotor subscore • Footprint analysis • Grid walking	• BBB: post-SCI weekly at 2–13 weeks (1–12 weeks post-implantation) • Footprint analysis: post SCI at 13 weeks • Grid walking: post-SCI at 13 weeks
Grosso et al. (53), USA	*n* = 40 Adult Female Sprague–Dawley rats Weight: 250–300 g	Traumatic SCI Complete right-sided lateral hemisection using microscissors Level: T8	• Liposomal clondronate/rolipram (*n* = 10) • Liposomal clondronate/rolipram/ChABC (*n* = 10) • ChABC (*n* = 10) • Vehicle (*n* = 10) **Rolipram:** 0.5 mg/kg/day delivered s.c. by mini-osmotic pump 0–7 days post-SCI **Clodronate:** encapsulated in liposomes (0.25 g/mL) and injected i.p. (2 mL per injection) on postinjury days 1, 3, and 6 **ChABC:** 1 mL of ChABC (20 U/mL) injected intraparenchymally into lesion center and 2 mm rostral and caudal to the lesion (7 days post-SCI) **Control:** Empty liposomes injected i.p. (days 1, 3, and 6 post-SCI) + DMSO delivered s.c. by osmotic minipump (0–7 days post-SCI) + 1 mL PBS injected into lesion center and 2 mm rostral and caudal to the lesion (7 days post-SCI)	Grid walking	Post-SCI on day 1 then weekly at 6 weeks
Iannotti et al. (51), USA	*n* = 40 Adult female Sprague–Dawley rats Weight: 220–250 g	Traumatic SCI Contusive injury by 10-g rod drop from height (12.5 mm) Level: T8	• Rolipram (*n* = 10) • Liposomal clodronate (*n* = 10) • Liposomal clodronate/rolipram (*n* = 10) • Vehicle (*n* = 10) **Rolipram:** 0.5 mg/kg/day delivered s.c. by mini-osmotic pump for 0–14 days post-SCI **Clodronate:** Injected i.p. (2 mL per injection) immediately after injury and 1,3, and 6 days post-SCI **Vehicle:** DMSO delivered s.c. by mini-osmotic pump for 0–14 days post-SCI	BBB locomotor score BBB locomotor subscore	Pre-SCI; post-SCI at day 1 then weekly at 4 weeks
Nazli et al. (58), Turkey	*n* = 24 Rabbit Weight: 2,400–3,500 g Level: Clamping distal to renal artery	Spinal cord ischemia Aortic occlusion with a vascular clamp distal to the renal artery	• Cilostazol (*n* = 8) • Vehicle (*n* = 8) • Sham-operated + vehicle (*n* = 8) **Cilostazol:** 30 mg/kg/day delivered orally via gavage for 3 days before the injury **Vehicle:** DMSO (30%)	Tarlov score	Post-ischemia at 24, 48, and 72 h
Nikulina et al. (54), USA	*n* = 12 Adult Long Evans Hooded rats Weight: 180–200 g	Traumatic SCI Right-sided lateral hemisection using iridectomy scissors (including dorsal columns bilaterally) Level: C3/4	• Rolipram 0.4 μmol/kg/h (*n* = 4) • Rolipram 0.8 μmol/kg/h (*n* = 3) • Vehicle (*n* = 5) **Rolipram:** 0.4 or 0.8 μmol/kg/h delivered s.c. by mini-osmotic pump from 14 to 24 days post-SCI **Vehicle:** DMSO (16%) delivered s.c. by mini-osmotic pump from 14 to 24 days post-SCI	Rearing test	Post-SCI at 8 weeks, tested on 3 consecutive days
Sahin et al. (59), Turkey	*n* = 24 Male Wistar albino rats Weight: 260–380 g Level: Clamping distal to renal artery	Spinal cord ischemia Aortic occlusion with a vascular clamp distal to the renal artery (45 min)	• Cilostazol (*n* = 8) • Vehicle (*n* = 8) • Sham-operated + vehicle (*n* = 8) **Cilostazol:** 20 mg/kg/day delivered orally for 3 days before the injury **Vehicle:** 1 mL of DMSO (30%) delivered orally for 3 days before the injury	Tarlov score	Pre-ischemia; post-ischemia at 48 h
Schaal et al. (31), USA	*n* = 24 Adult female Fisher rats Weight: 180–200 g	Traumatic SCI Contusive injury by 10-g rod drop from height (12.5 mm) Level: T8	Rolipram (*n* = 8) • Vehicle (*n* = 8) • Injury only (*n* = 8) **Rolipram:** 1.0 mg/kg delivered i.v. 1 h post-SCI **Vehicle:** 10% ethanol in 0.9% physiological saline delivered i.v. 1 h post-SCI	BBB locomotor score BBB locomotor subscore	Post-SCI weekly at 1–6 weeks
Yamamoto et al. (61), Japan	*n* = 40 Adult (12–14 weeks) male Wistar rats Weight: 250–270 g	Chronic compression—sheet of expanding polymer inserted (constant size after 48–72 h) Level: C5/6	• Sham-operated + vehicle (*n* = 7) • Sham-operated + cilostazol (*n* = 7) • Polymer sheet implantation (*n* = 13) • Polymer sheet implantation + cilostazol (*n* = 13; unclear whether all mice were used in neurobehavioral assessment) **Cilostazol:** 30 mg/kg/day orally once daily for 0 to 175 days post-SCI **Vehicle:** 0.5% carboxymethyl cellulose sodium salt solution delivered orally once daily for 0 to 175 days post-SCI **Sham:** polymer sheet was placed underneath the laminae momentarily and then removed	Grip strength Voluntary exercise Forced locomotion capability	Voluntary exercise: pre-SCI; post-SCI Grip strength: pre-SCI; post-SCI twice weekly for 25 weeks Forced locomotion capability: pre-SCI; post-SCI weekly at 1–25 weeks
Yin et al. (52), China	*n* = 36 Adult female Sprague–Dawley rats Weight: 200–220 g	Traumatic SCI Contusive injury by 10-g rod drop from height (25 mm) Level: T9/10	• Rolipram (*n* = 8) • Methylprednisolone (*n* = 8) • Rolipram + Methylprednisolone (*n* = 8) (unclear whether all mice were used in neurobehavioral assessment) • Sham-operated (*n* = 4) • Vehicle (*n* = 8) **Rolipram:** 0.5 mg/kg/day delivered by mini-osmotic pump for 0–14 days post-SCI **Methylprednisolone sodium succinate:** 30 mg/kg delivered IV immediately post-SCI **Vehicle:** injected daily for 0–14 dayspost-SCI	BBB locomotor score Grid walking	BBB: pre-SCI; post-SCI at 24 h and 3 days post-injury, then weekly at 1–8 weeks. Grid walking: post-SCI at 8 weeks
Bretzner et al. (64), Canada	*n* = 47 Adult male Sprague–Dawley rats Weight: 300–400 g	Traumatic SCI Dorsolateral funiculus crushed with custom fine surgical forceps 2 mm from the surface for 20 s Level: C4-5	• Rolipram (*n* = 4;4;4) • Rolipram + OEC (*n* = 7;4;7) • OEC (*n* = 5;4;8) • Db-cAMP (*n* = 4;4;4) • Db-cAMP + OEC (*n* = 10;4;4) • OEC (*n* = 7;4;7) • Vehicle (*n* = 8;6;4; cylinder; reaching; sensory tests) **Rolipram:** 4 μmol/kg/h delivered s.c. by mini-osmotic pump for 0 to 14 days post-SCI **Dibutyryl cAMP:** 0.5 μg/μl/h delivered by mini-osmotic pump in the vicinity of the red nucleus for 0–14 days post-SCI **OEC (Olfactory ensheathing cells):** 150,000–180,000 lamina propria-derived OECs injected 1 mm rostral and caudal to the lesion site **Vehicle:** DMEM/F-12 delivered by mini-osmotic pump and injections using the same methods as non-vehicle treatment groups	Thermal allodynia Rearing test Food-pellet reaching test	Pre-SCI; post-SCI weekly at 1–4 weeks
Pearse et al. (46), USA	*n* = 144 Adult female Fischer rats Weight: 160–180 g	Traumatic SCI “Moderate” contusive injury using impactor Level: T8	• Acute rolipram (*n* = 12) • Acute rolipram + SC transplant (*n* = 12) • Acute rolipram + SC transplant and db-cAMP (*n* = 12) • Delayed rolipram + SC transplant and db-cAMP (*n* = 12) • Schwann cell (SC) transplant (*n* = 12) • SC transplant + db-cAMP (*n* = 12) • Vehicle (*n* = 12) **Acute rolipram:** 0.5 mg/kg/day delivered s.c. by mini-osmotic pump for 0–14 days post-SCI **Delayed rolipram:** 0.5 mg/kg/day delivered s.c. by mini-osmotic pump for 7 to 21 days post-SCI **Dibutyryl cAMP:** 0.25 μl of 50 mM db-cAMP injected into the spinal cord rostral and caudal to the SC graft at a depth of 0.5 mm, 1 week post-SCI **Schwann cell (SC) transplant:** 2 × 10^6^ SCs in 6 μl DMEM-F12 medium were injected into the contused area, 1 week post-SCI **Vehicle:** DMSO delivered by mini-osmotic pump and injected with rolipram and cAMP	BBB locomotor score BBB locomotor subscore Grid walking Footprint analysis	BBB score and subscore: pre-SCI; post-SCI weekly at 1–8 weeks Grid walking: post-SCI at 8 weeks Footprint analysis: post-SCI at 8 weeks
Wang et al. (47), USA	*n* = 49 Adult (11–12 weeks) Female Sprague–Dawley rats Weight: 250–270 g	Traumatic SCI Contusive injury using impactor (rapid displacement of cord surface by 1.1. mm for 20 ms) Level: T8	• Rolipram (*n* = 10) • Rolipram + Nogo-66 receptor protein (*n* = 8) • Nogo-66 receptor protein (*n* = 16) • Vehicle (*n* = 15) **Rolipram:** 1.2 mg/kg/day delivered s.c. by mini-osmotic pump from day 3 to 31 post-SCI **Nogo-66 receptor protein [NgR(310)ecto-FC]:** 0.29 mg/kg/day delivered intracerebroventricularly by mini-osmotic pump from day 3 to day 31 post-SCI **Vehicle:** PBS delivered s.c. and intracerebroventricularly using the same method as rolipram and Nogo-66 groups	BBB locomotor score	Post-SCI at 2 days, then weekly at 1–5 weeks, then at 49 days
Downing et al. (57), USA	*n* = 15 Adult female rats (athymic, National Cancer Institute) Weight: 170–243 g	Traumatic SCI Complete right-sided lateral hemisection using ‘a fine scalpel blade' Level: C4-6	• Low-dose rolipram patch (*n* = 3) • High-dose rolipram patch (*n* = 4) • Vehicle patch (*n* = 4) • Hemisection with no patch (*n* = 4) **Rolipram:** microfibrous patch measuring 0.5 × 0.3 cm with 3.1 g/cm^2^ (low dose) or 62.5 g/cm^2^ (high dose) of rolipram implanted at the time of SCI **Vehicle patch:** microfibrous patch measuring 0.5 × 0.3 cm with no rolipram	Martinez forelimb score	Post-SCI at 2 days, then weekly at 1, 2, 3, 4, 5, 6, and 8 weeks
Dai et al. (63), USA	*n* = 52 Adult (6 weeks) Female Sprague-Dawley rats	Traumatic SCI Surgical right-sided over-hemisection Level: C4-5	Standard housing • Sham (*n* = 5) • Hemisection only (*n* = 6) • Hemisection + vehicle (*n* = 6) • Hemisection + rolipram (*n* = 9) Enriched housing • Sham (*n* = 5) • Hemisection only (*n* = 6) • Hemisection + vehicle (*n* = 5) • Hemisection + rolipram (*n* = 10) **Rolipram:** 0.4 μmole/kg/h delivered s.c. by mini-osmotic pump for 0–10 days post-SCI **Vehicle:** DMSO (15%) delivered using the same method as the rolipram group	Skilled target reaching Grid walk Vertical exploration CatWalk gait analysis	Reaching, gait, and grid walk assessment weekly (1–4 weeks) Vertical exploration assessed at 4 weeks
Koopmans et al. (48), Netherlands	*n* = 74 Adult (12 weeks) Male Lewis rats	Traumatic SCI Contusive injury using impactor (12.5 g cm) Level: T10	• Rolipram (*n* = 12) • Thalidomide (*n* = 12) • Thalidomide + rolipram (*n* = 20) • Vehicle (*n* = 20) • Sham (*n* = 4) • No lesion (*n* = 4) **Rolipram:** i.p. injection (3 mg/kg) delivered immediately post-SCI **Thalidomide:** i.p. injection (100 mg/kg) delivered immediately post-SCI **Vehicle:** i.p. injection of 1% methylcellulose, 0.1% Tween-80 in sterile saline, delivered immediately post-SCI	BBB locomotor score and subscore CatWalk gait analysis	BBB score and subscore assessed pre-SCI then at 1, 3, and 5 days post-SCI, then weekly for 1–6 weeks CatWalk gait analysis performed pre-SCI then at 1 and 6 weeks post-SCI
Kurtoglu et al. (60), Turkey	*n* = 24 Adult male Sprague-Dawley rats Weight: 290–320 g Level: Clamping distal to renal artery	Spinal cord ischemia Aortic occlusion with a vascular clamp distal to the renal artery (45 min)	• Cilostazol (*n* = 8) • Vehicle (*n* = 8) • Sham (*n* = 8) **Cilostazol:** 20 mg/kg per day administered by i.p. injection for 3 days pre-injury **Vehicle:** DMSO administered by i.p. injection for 3 days pre-injury	Modified Tarlov score	Assessed 48 h post-ischemia
Nout et al. (49), USA	*n* = 45 Adult male rats (71 ± 2 days)	Traumatic SCI Contusive injury using impactor (25 g cm) Level: T11	• Rolipram + cAMP (*n* = 12) • GRP cell transplant (*n* = 11) • Rolipram + GRP cAMP (*n* = 11) • Vehicle (*n* = 11) **Rolipram:** 0.5 mg/kg/day delivered s.c. by mini-osmotic pump from 0 to 2 weeks post-SCI **GRP cell transplant:** 2–3 × 10^6^ GRP cells in 10 μl PBS injected into three sites in the lesion region at 9 days post-SCI **cAMP:** 2 × 0.25 μl 50 mM injections at 9 days post-SCI **Vehicle:** 0.45% NaCl in DMSO delivered s.c. using the same method as rolipram, GRP, and cAMP injections.	BBB locomotor score Cage activity assessment	BBB score assessed at 1, 2, 7, 10, 16, 22, 30, 37, 44, 51, 58, 65, 72, 79, and 86 days post-SCI. Cage activity recorded on days 1, 5–8, 10–15, 21, 28, 35, 42, 49, 56, 63, 70, 77, and 84 post-SCI.
Sharp et al., USA^62^	*n* = 27 Adult Female Fisher rats	Traumatic SCI Contusive injury using impactor (10 g from 12.5 mm height, 2 mm rod diameter) Level: T8-9	Squad 1: • Rolipram (*n* = 4) • Rolipram + Schwann cell injection (*n* = 6) • Vehicle (*n* = 6) Squad 2: • Rolipram (*n* = 6) • Schwann cell injection (*n* = 7) • Rolipram + Schwann cell injection + db-cAMP (*n* = 7) • Vehicle (*n* = 12) **Rolipram:** 0.5 mg/kg/day delivered s.c. by mini-osmotic pumps for 0–14 days post-SCI **Schwann cell transplant:** 2 × 10^6^ Schwann cells in 6 μl vehicle at the center of the SCI lesion at a depth of 1 mm, delivered 7 days post-SCI **Db-cAMP:** 0.25 μl of 50 mM db-cAMP injected at two sites 4 mm rostral and two sites 4 mm caudal to the lesion center. Delivered at 7 days post-SCI. **Vehicle:** DMEM delivered by injection using the same method as the cell transplant and cAMP. Empty mini-osmotic pumps were implanted.	BBB locomotor score BBB locomotor subscore Grid walking Ladder beam task Footprint analysis Kinematic analysis	Squad 1: BBB analysis: days 16, 23, 29, 38, 43, 49, 60, 64, and 70 post-SCI Grid walk: day 23, 25, 32, 36, 42, 50, 58, and 64 post-SCI Footprint analysis: pre-SCI, then day 21, 31, 39, 45, 59, and 66 post-SCI Ladder beam: 65 days post-SCI Video kinematic analysis: day 67 post-SCI Squad 2: BBB analysis: day 14, 16, 23, 29, 31, 38, 43, 49, 59, 64, and 69 post-SCI Grid walk: day 23, 32, 36, 42, and 50 post-SCI Footprint analysis: day 21, 24, 38, 44, 59, and 66 post-SCI Ladder beam: day 65 post-SCI Video kinematic analysis: day 67 post-SCI
Moradi et al. (56), Iran	*N* = 50 Male rats Weight: 240–260 g	Traumatic SCI Contusive injury using aneurysmal clip (YASARGIL^®^ Aneurysm clip system) Level: T9	• Vehicle (*n* = 10) • Sham (*n* = 10) • Low-dose roflumilast (*n* = 10) • Medium-dose roflumilast (*n* = 10) • High-dose roflumilast (*n* = 10) **Vehicle:** Saline 0.9% **Low dose:** Single dose of 0.25 mg/kg roflumilast before induction of SCI **Medium dose:** Single dose 0.5 mg/kg roflumilast before induction of SCI **High dose:** Single dose 1 mg/kg roflumilast before induction of SCI	BBB locomotor score	Assessed at baseline then on days 1, 3, 7, 14, 21, and 28 post-SCI.

i.p., intraperitoneal injection; s.c., subcutaneous injection, i.v. intravenous injection.

### Risk of bias

The allocation sequence was only adequately generated and applied in 10 of 22 studies. The remaining studies may have been randomized but did not describe their allocation sequence. One study described random housing of animals (63). In total, nine studies stated that group neurobehavioral characteristics were similar to baseline (31, 44, 48, 49, 52, 60–63). No studies stated whether animals were randomly selected for assessment or whether group allocation was adequately concealed. The outcome assessor was blinded to treatment groups in 16 studies (31, 44, 45, 47, 48, 50, 51, 53–55, 57–59, 62–64). Comprehensive risk-of-bias assessment scores are provided in Supplementary material 3.

### What is the impact of PDE inhibition on neurobehavioral outcomes?

The findings of each included study are summarized in Table 6.

**Table 6:** Statistics analysis and main conclusions of included studies.

**References, location**	**Statistical analysis**	**Main conclusions**
Bao et al. (55), Canada	• One-way ANOVA • Student–Newman–Keuls (SNK) testing	• Treatment with 0.5 mg/kg PDE4-I (IC486051) improved BBB scores significantly from 4 to 8 weeks after SCI (*p* = 0.03-0.05 vs. vehicle) with a mean difference of 1.3 points. • A significant effect of 1.0 mg/kg PDE4-I treatment was detected by ANOVA (*p* < 0.001 vs. vehicle), but the mean values did not differ significantly from the control group. • Mechanical allodynia elicited from the hind paw was significantly lower with PDE4-I treatment from 4 to 6 weeks post-SCI (0.5 mg/kg, *p* < 0.001; 1.0 mg/kg, *p* < 0.001 vs. vehicle).
Beaumont et al. (44), USA	• Independent *t*-tests • Spearman rank correlations	• The BBB scores of the rolipram-treated rats (14.2 ± 1.8) and vehicle-treated rats (13.4 ± 0.8) were similar (*p* = 0.07). • In grid walk assessment, rolipram-treated rats had a higher percentage of hindlimb steps without footfall errors (*p* = 0.05 vs. vehicle). There was no significant difference in total number of steps or percentage of forelimb steps without footfall errors.
Costa et al. [45, Portugal	• Mann–Whitney *U*-test • 2-way ANOVA (General Linear Model)	• BBB scores of rolipram-treated rats were significantly higher than vehicle-treated rats at all timepoints from 7 days post-SCI (*p* = 0.05). • Rolipram-treated rats had significantly higher beam walk scores at all timepoints from 3 weeks post-SCI (*p* = < 0.05). • After 8 weeks, 3D hindlimb kinematics analysis found significantly decreased external rotation during the stance phase at initial contact in rolipram-treated animals (*p* = 0.027 vs. vehicle).
Flora et al. (50), USA	• Mixed-factorial (repeat measures) ANOVA • Turkey–Kramer test • One-way ANOVA • Bonferroni *post-hoc* test	• BBB scores and subscores of animals treated with rolipram in addition to stem cells (SC) were greater than those treated with GFP SC alone (*p* < 0.05). • In footprint analysis, animals treated with rolipram and D15A SCs had significantly less foot exrotation (*p* < 0.001), the narrower base of support (*p* < 0.001), and fewer footfall errors on grid walking (*p* < 0.001) compared with vehicle and SC only groups.
Grosso et al. (53), USA	• Repeated-measures analysis of variance • Tukey's *post-hoc* test	• On-grid walking assessment, clodronate/rolipram-treated rats had significantly lower % footfalls than the control group at day 28 (*p* < 0.05). • Clodronate/rolipram/ChABC-treated rats had significantly lower % footfalls than the control group from day 14 onwards (day 14, *p* < 0.05; day 21–35, *p* < 0.01; day 42, *p* < 0.001). • There were no statistically significant differences between the clodronate/rolipram group and clodronate/rolipram/ChABC group on grid walking assessment at any timepoint.
Iannotti et al. (51), USA	• Repeated-measures ANOVA • Tukey's *post-hoc* test	• 1 week post-SCI animals receiving the clodronate and/or rolipram treatment demonstrated significant improvement in hindlimb locomotion compared with controls (clodronate group or rolipram group *p* < 0.05; clodronate/rolipram group, *p* < 0.001). • 4 weeks post-SCI, animals receiving the clodronate and/or rolipram treatment demonstrated significant improvement in inter-limb coordination, toe clearance, and paw placement compared with controls (clodronate group or rolipram group, *p* < 0.05; clodronate/rolipram group, *p* < 0.01). • No significant differences in BBB locomotor scores were observed between combined treatment and each of the single treatment groups, or between either of the single drug treatment groups, at any time point. • In BBB subscore analysis, 1 week after injury, significant improvements in fine details of hindlimb function were seen after delivery of clodronate and/or rolipram, compared with controls (clodronate group or rolipram group *p* < 0.05; clodronate/rolipram group, *p* < 0.05). • By 4 weeks post-injury, significant improvements in BBB subscores were observed after clodronate and/or rolipram treatment, as compared to controls (*p* < 0.001). At each time-point, significant differences were also present between the combined drug treatment group and each single drug treatment group (*p* < 0.05).
Nazli et al. (58), Turkey	• Shapiro–Wilk test • Kruskal–Wallis test • Mann–Whitney *U*-test • Friedman test	• Median Tarlov scores postoperatively at all intervals (24, 48, and 72 h) were significantly higher in the cilostazol group than in the ischemia-reperfusion-only group (*p* < 0.001).
Nikulina et al. (54), USA	• Not stated	• Right forelimbs were impaired by cord hemisection. When assessing rearing, the number of forelimb contacts that were dorsal (wrong) was significantly lower in rats treated with rolipram (35% with rolipram treatment vs. 75% with vehicle treatment, *p* < 0.05). • Rolipram-treated animals raise the injured limb more frequently above the horizontal plane (76% for the rolipram group, 56% for the vehicle group), suggesting greater proximal forelimb control.
Sahin et al. (59), Turkey	• Not stated	• Mean (±SD) Tarlov scores at 48 h post-ischemia were 3.66 ± 0.40 in the cilostazol-treated group and 2.32 ± 0.80 in the ischemia-only group (*p* = 0.08).
Schaal et al. (31), USA	• Repeated-measures one-way ANOVA	• No significant difference in BBB score was observed between any groups in the first 3 weeks post-injury (*p* < 0.05). • Rolipram-treated animals had significantly higher BBB scores at 4, 5, and 6 weeks, vs. SCI only and SCI + vehicle groups (*p* < 0.5). • In BBB subscoring, no significant intergroup differences were found at any timepoints.
Yamamoto et al. (61), Japan	• Repeated-measures one-way ANOVA	• No significant difference in left and right forepaw grip strength was observed between the compression + cilostazol group and either sham group (sham + vehicle or sham + cilostazol). Grip strength decreased significantly in the compression + vehicle group (*p* < 0.05 vs. sham + vehicle) from 7 weeks onwards. • Voluntary exercise decreased gradually post-SCI with no significant intergroup differences. • There was no significant difference in forced locomotion capability between the compression + cilostazol group and the sham groups. Forced locomotion capability decreased significantly in the compression + vehicle group (*p* < 0.05 vs. sham + vehicle).
Yin et al. (52), China	• One-way ANOVA • Newman–Keuls' multiple comparison tests	• Mice treated with rolipram or methylprednisolone (MP) alone had BBB scores and grid walking scores that were not significantly different from the vehicle group at any timepoint. • Mice treated with a combination of rolipram and MP had significantly higher BBB scores at all timepoints from 3 weeks post-SCI (*p* < 0.01 vs. vehicle; *p* < 0.05 vs. rolipram group or MP group, at 3–8 weeks post-SCI). • Grid walking test showed that the percentages of missteps in the combined rolipram + MP group were significantly lower than the control group (*p* < 0.01).
Bretzner et al. (64), Canada	• Repeated-measures two-way ANOVA • Kruskal–Wallis test • Chi-squared test	• With thermal stimulation, withdrawal latency in the left forepaw (impaired by hemisection) was significantly shorter in rats treated with olfactory unsheathing cells (OEC) at 3 weeks (*p* < 0.05 vs. vehicle-treated group) and in rats treated with OEC + rolipram at 4 weeks (*p* < 0.05 vs. vehicle-treated group). In the right forepaw, latency was significantly reduced with OEC treatment when compared with rolipram or OEC + rolipram groups (*p* < 0.05). • Only animals treated with a combination of OEC + rolipram had significantly greater usage of the injured forearm in rearing tests (left + both forelimb use/left + right + both forelimb use = 59%, *p* < 0.05 vs. vehicle-treated group). • There were no significant differences between groups in the food-pellet-reaching task.
Pearse et al. (46), USA	• ANOVA • Tukey–Kramer test	• Animals in the acute rolipram + stem cells (SC) + dc-cAMP group had significantly greater BBB scores than vehicle-treated animals at 3–8 weeks post-SCI (*p* < 0.05) and significantly greater BBB subscores than the vehicle group at 2–8 weeks post-SCI (*p* < 0.05). • Animals in the acute rolipram-only group had significantly greater BBB scores than vehicle-treated animals at 4 and 5 weeks post-SCI (*p* < 0.05), and significantly greater BBB subscores than the vehicle group at 4–8 weeks post-SCI. • All other groups were not significantly different from the control group, except for the BBB subscores of the SC + db-cAMP group at 7 and 8 weeks post-SCI. • In footprint analysis, animals in the acute rolipram only, acute rolipram + SC, and acute rolipram + SC + db-cAMP all showed significantly less foot exorotation than the control group (*p* < 0.01, *p* < 0.05, and *p* < 0.01, respectively), a narrower base of support (*p* < 0.01), and fewer footfall errors on the irregular grid walk test (*p* < 0.05, *p* < 0.01, and *p* < 0.01, respectively).
Wang et al. (47), USA	• ANOVA • Repeated-measures ANOVA	• The NgR + rolipram group had BBB scores that were indistinguishable from the NgR-only group but were significantly greater than the vehicle group at day 49 post-SCI (*p* ≤ 0.05). • BBB locomotor scores showed a significant improvement in the NgR(310)ecto-Fc–treated group (difference between groups *p* ≤ 0.05 and across time *p* ≤ 0.001). • Animals receiving rolipram-only treatment had BBB scores that were not significantly different from the vehicle-treated group at any timepoint.
Downing et al. (57), USA	• Repeated-measures two-way ANOVA • Tukey's *post-hoc* test	• Martinez forelimb open-field scores showed that animals treated with low-dose rolipram patches score significantly higher, from weeks 1 through 4, 6, and 8, when compared to all other groups. Animals treated with patches loaded with 20 times more rolipram showed no significant differences with respect to untreated animals. • In particular movement scoring, low-dose rolipram-treated animals scored significantly higher than all other groups after 1 week post-SCI. No other scores were significantly different. • 100% of rats treated with low-dose rolipram patches displayed “consistent” or “frequent” coordination behaviors (assessed at 8 weeks, *n* = 3). Animals treated with high-dose rolipram patches displayed no coordination behaviors (*n* = 4). For animals treated with blank patches, 75% displayed “frequent” or “occasional” coordination, and 25% showed no coordination (*n* = 4); 75% of untreated animals displayed no coordination, while 25% were “occasionally” coordinated (*n* = 4).
Dai et al. (63), USA	• Repeated-measures ANOVA • Three-way ANOVA • Two-way ANOVA • Bonferonni's *post-hoc* analysis	• In the grid walk assessment, there was no significant reduction in errors made in animals treated with rolipram, and there was no significant interaction between enriched environments and daily training and rolipram. • In vertical exploration testing, there was no significant increase in right forelimb contacts after rolipram and/or enriched environments with daily training. • No CatWalk gait analysis measures were significantly affected by rolipram or enriched environments with daily training.
Koopmans et al. (48), Netherlands^=^	• Repeated-measures ANOVA • One-way ANOVA • Bonferroni *post-hoc* test	• BBB scores and subscores of rolipram-treated animals were not significantly different from vehicle-treated animals. • BBB scores in combined rolipram/thalidomide groups were significantly higher than all other groups at 7 and 42 days post-SCI (*p* < 0.01). • BBB subscores in combined rolipram/thalidomide groups were significantly higher than all other groups at 14, 21, and 42 days post-SCI (*p* < 0.01). • The CatWalk regularity index (RI) at 42 days post-SCI found that rolipram + thalidomide-treated animals returned to pre-test scores, whereas all other groups were significantly different from pre-test scores (*p* < 0.05). • The Catwalk-based BBB score showed a significant improvement in the recovery of locomotor performance in only the combined drug treatment group by 42 days post-SCI (*p* < 0.01).
Kurtoglu et al. (60), Turkey	• Tukey's multiple comparison test • One-way ANOVA	• There was no significant difference in Tarlov scores between injured groups at any timepoint.
Nout et al. (49), USA	• Repeated-measures ANOVA • Repeated-measures ANCOVA • One-way ANOVA • Holm–Sidak procedure for pairwise multiple comparison *post-hoc* test	• There was no significant difference in BBB scores between groups at any timepoint. • There was no significant difference in cage activity between groups at any timepoint.
Sharp et al. (62), USA	• Repeated-measures ANOVA • One-way ANOVA • Tukey's multiple comparison test	• There was no significant difference in BBB scores or subscores between groups at any timepoint (analyzed in squad 1, 2, and 1+2 combined) • In footprint analysis using paint, there was no significant difference between groups at any timepoint in paw rotation, base of support, or stride length at any timepoint (analyzed in squad 1, 2, and 1+2 combined). • In video kinematic analysis there were no significant differences between groups at any timepoint in foot exrotation, base of support or stride length (analyzed in squad 1, 2, and 1+2 combined). • In grid walk testing, the animals that received Schwann cells only exhibited significantly fewer footfall errors than all other groups. The group that received Rolipram + Schwann cells + db-cAMP combined had significantly fewer errors than the rolipram-treated group, but did not differ from the vehicle-treated group (in analysis of squad 1+2 combined; there were no significant differences when analyzing each group individually). • In ladder beam assessment there was no significant difference in number of hindlimb footfall errors between groups (analyzed in squad 1, 2, and 1+2 combined).
Moradi et al. (56), Iran	• General linear model repeated-measures analysis • Tukey's multiple comparison test • One-way ANOVA • Repeated-measures ANOVA	• BBB scores significantly higher for all groups compared with sham at all timepoints (*p* < 0.05). • BBB scores significantly improved for all groups that received roflumilast 28 days post-lesion (*p* < 0.05). • BBB score improved most for group treated with 0.5 mg/kg roflumilast. • BBB scores for 0.5 and 1 mg/kg groups significantly higher than 0.25 mg/kg group treated with roflumilast (*p* < 0.001 and *p* = 0.03, respectively). • BBB scores for 0.5 and 1 mg/kg group statistically similar (*p* = 0.64).

#### Basso, Beattie, and Bresnahan (BBB) locomotor score

Of the 13 studies that assessed BBB scores, 11 involved rolipram-treated animals (31, 44–52, 55, 56, 62), one involved PDE4 inhibitor-treated animals (55), and one involved roflumilast-treated animals (56).

Of nine studies involving animals treated exclusively with rolipram, three found that rolipram-treated animals had significantly higher BBB scores than vehicle-treated animals (31, 45, 46). This was observed from 7 days post-SCI by Costa et al. (45), from 4 weeks post-SCI by Schaal et al. (31), and at 4 and 5 weeks post-SCI (but not at 6–8 weeks) by Pearse et al. (46). In total, six studies found that the BBB scores of animals treated with rolipram alone were not significantly different to vehicle treatment groups (44, 47, 48, 51, 52, 62); two studies assessing BBB score did not include a group treated solely with rolipram (49, 50).

In total, four studies found BBB scores were significantly higher than vehicle-treated animals when rolipram was combined with stem cells with cAMP (from 3 weeks post-SCI with rolipram delivered at the time of injury) (46); Nogo-66 receptor protein (at 49 days post-SCI) (47); methylprednisolone (from 3 weeks post-SCI) (52); thalidomide (at 7 and 42 days post-SCI) (48); and stem cells with a green fluorescent protein (from 2 weeks post-SCI) (50). In contrast, four studies found BBB scores were not significantly different to vehicle-treated animals when rolipram treatment combined with clodronate (51), Schwann cells (with or without cAMP) (62), and cAMP (with or without glial restricted precursor cells) (49).

Using PDE4-I, a selective PDE4 inhibitor, Bao et al. found treatment with 0.5 mg/kg improved BBB scores significantly from 4 to 8 weeks post-SCI (55). Moradi et al. found that treatment with 0.25, 0.5, and 1 mg/kg of roflumilast all improved the BBB score significantly compared with the vehicle 28 days post-SCI (12).

#### Basso, Beattie, and Bresnahan (BBB) locomotor subscore

In total, six studies assessed BBB subscore, all of which involved rolipram-treated rats; five studies involved animals treated exclusively with rolipram, two of which found animals treated with rolipram alone had significantly higher BBB scores than vehicle-treated animals. Significant benefit was observed from 1 week post-SCI by Iannoti et al. (51) and from 4 weeks post-SCI by Pearse et al. (with acute, not delayed rolipram administration) (46); In total, three studies found that the BBB subscores of animals treated exclusively with rolipram were not significantly different from vehicle treatment groups (31, 48, 62). One study assessing BBB subscore did not include a group treated solely with rolipram (50).

In total, four studies found BBB subscores were significantly higher than vehicle-treated animals when combining rolipram with clondronate (from 1 week post-SCI) (51), stem cells and cAMP (from 2 weeks post-SCI) (46), thalidomide (at 14, 21, and 42 days post-SCI) (48), and stem cells and green fluorescent protein (at 4 and 8–12 weeks post-SCI, or with GFP-D15A at 3–5 and 7–12 weeks post-SCI) (50). One study found BBB subscores were not significantly different than vehicle-treated animals when rolipram was combined with Schwann cells (with or without cAMP) (62).

#### Modified Tarlov score

Three studies used the modified Tarlov score in their assessments (58–60), each using a spinal cord ischemia injury model and cilostazol treatment. Nazli et al. studied rabbits and found that median Tarlov scores were significantly higher in the cilostazol group than in the ischemia–reperfusion-only group at all post-ischemia intervals (1–3 days) (58). In contrast, Sahin et al. reported that mean Tarlov scores in the cilostazol group were similar to the ischemia group (assessed at 2 days only, *p* = 0.08) in rats (59). Kurtoglu et al. studied rats and found that there was no significant difference in Tarlov scores between injured groups at any timepoint (60). In their study, sham group rats were subjected to laparotomy without aortic occlusion. Control group rats were pre-treated with intraperitoneal dimethyl sulfoxide while the cilostazol group rats received intraperitoneal cilostazol (20 mg/kg/day) for 3 days before the induction of ischemia. Ischemia was induced by clamping of the infrarenal aorta.

#### Grid walking

In total, seven studies assessed grid walk performance, all involving rolipram-treated rats (44, 46, 50, 52, 53, 61, 62). A regular grid was used in three studies (44, 53, 63), and an irregular grid was used in four studies (46, 50, 52, 62). Two studies found that rats treated exclusively with rolipram had significantly fewer footfall errors than vehicle-treated rats (44, 46). Beaumont et al. reported that rolipram-treated rats had a higher percentage of hindlimb steps without footfall errors, although no significant difference was found in the total number of steps or percentage of forelimb steps without footfall errors (44). Three studies found that in grid walk testing, there was no significant difference in the rates of footfall errors between rolipram- and vehicle-treated groups (52, 62, 63).

In total, five studies assessed grid walking after rolipram combined with other treatments (46, 50, 52, 53, 62); two of these studies did not include a group treated exclusively with rolipram (50, 53). These studies found that, when compared to vehicle-treated rats, grid walk footfall errors occurred at significantly lower rates when rolipram was combined with methylprednisolone (52); stem cells (with acute, not delayed rolipram administration, and with or without dc-cAMP) (46); D15A stem cells (50); and clodronate (with or without chondroitinase) (53). Sharp et al. found rats that received a combination of rolipram, Schwann cells, and db-cAMP had significantly fewer errors than the rolipram-treated group, but did not differ from the vehicle-treated group (62).

#### Vertical exploration/rearing

Three studies assessed vertical exploration behaviors (54, 63, 64). Dai et al. found that when rearing, there was no significant difference in right forelimb wall contacts between rolipram and vehicle-treated animals (63). Bretzner et al. found that there was no significant difference in forelimb usage between rolipram and vehicle-animal-treated animals in vertical exploration, though animals treated with a combination of rolipram and olfactory ensheathing cells demonstrated significantly greater usage of the injured forelimb (64). Nikulina et al. found that animals treated with embryonic spinal cord tissue and rolipram had significantly fewer incorrect (dorsal) forelimb contacts and raised the injured limb more frequently above the horizontal plane than animals receiving transplant alone (54).

#### Footprint and gait analysis

The footprint assessment method was variable, involving video-based kinematic analysis (62, 63), paint (62), or ink (50), or an unspecified technique (46). Multiple studies found no significant difference between animals treated exclusively with rolipram- and vehicle-treated animals when measuring foot exrotation (46, 62), base of support (46, 62, 63), or stride length (46, 62, 63). When rolipram was combined with stem cells and db-cAMP significantly less foot exrotation was observed (46), and when combined with D15A stem cells significantly improved base of support and stride length were observed (50). Compared with vehicle treatment, no significant difference in foot exrotation, base of support, or stride length was found when combining rolipram with Schwann cells, with or without cAMP (62).

Assessing gait using 3D video kinematic analysis, Costa et al. found significantly decreased hindlimb exrotation during the stance phase at initial contact in rolipram-treated animals (assessed at 8 weeks post-SCI) (45). Using CatWalk video analysis, Dai et al. identified no difference in standtime or swingtime when comparing rolipram and vehicle-treated animals (63).

Also using CatWalk video analysis, Koopmans et al. reported that neither gait coordination (quantified using the CatWalk regularity index) nor an integrated Catwalk-based BBB score was significantly different when comparing vehicle and rolipram-treated animals. Both metrics were significantly improved by 42 days post-SCI in rats treated with a combination of rolipram and thalidomide (compared with vehicle-treated rats) (48).

#### Reaching tasks

In total, two studies assessed animal reaching capabilities; there were no significant differences between rolipram and vehicle-treated animals in their ability to reach food through a small opening (with or without combination with db-cAMP and/or olfactory ensheathing cells) (64), or from vertical shelves (63).

#### Beam walking

In total, two studies included beam walking assessments (45, 62). Costa et al. reported that from 3 weeks post-SCI, rolipram-treated rats had significantly higher beam walk scores (i.e., fewer foot slips) than vehicle-treated rats (45). In ladder beam assessment, Sharp et al. found no significant difference in the number of hindlimb footfall errors between vehicle- and rolipram-treated mice (including mice treated with a combination of rolipram and Schwann cells, with or without cAMP) (62).

#### Allodynia

Bao et al. found mechanical allodynia elicited from hindpaws was significantly lower with PDE4 inhibitor treatment from 4 weeks post-SCI (55). Bretzner et al. reported that when applying thermal stimulation, the withdrawal latency in injured forepaws was not significantly different in rolipram-treated rats compared with vehicle-treated rats, but was significantly shorter in rats treated with rolipram and olfactory ensheathing cells from 4 weeks post-SCI (64).

#### Voluntary activity

Two studies assessed rates of voluntary activity (49, 61). No significant difference was found between rolipram-treated and vehicle-treated rats in voluntary movement inside the animal housing (with or without cAMP and/or glial restricted precursor cell transplant) (49), or between cilostazol-treated and vehicle-treated rats in voluntary exercise on a wheel inside the animal housing (61).

#### Additional measures

Yamamoto et al. found there was no significant difference in forced locomotor capability or forepaw grip strength between rats that received chronic compression and cilostazol treatment and rats that received a sham treatment. Forced locomotor capability and grip strength were significantly higher in sham-treated rats than those receiving chronic compression and vehicle treatment (61).

Downing et al. found, when compared to vehicle treatment, animals treated with low-dose rolipram patches had significantly higher Martinez forelimb open-field scores at 1–4, 6, and 8 weeks post-SCI, with significantly higher articular movement scores from 1 week post-SCI. There were no differences in scores between animals treated with high-dose rolipram and vehicle treatment. Animals treated with low-dose rolipram patches displayed the highest rates of coordinated forelimb–hindlimb behaviors of any group, while animals treated with high-dose rolipram patches displayed fewer coordinated behaviors than animals treated with unmedicated patches (57).

## Discussion

The objective of this systematic review was to synthesize current literature evidence concerning the effect of PDE inhibitors on neurobehavioral outcomes in preclinical models of traumatic and non-traumatic SCI. Overall, PDE inhibitors were associated with statistically significant improvements in neurobehavioral outcomes in a majority of included studies. However, evidence was inconsistent with a high risk of bias, including inadequate or unreported allocation sequence and a lack of standardized methodologies.

### Proposed mechanism of action

Mechanistic explanation for these results include rolipram antagonizing SCI-induced PDE4B1 and PDE4A5 production, PDE4A5 phosphorylation, and MCP-1 expression, reducing immune cell infiltration and preventing post-injury reduction in IL-10 (31). Furthermore, Bao et al. have demonstrated that the PDE4 inhibitor PDE4-I has anti-inflammatory and anti-oxidative effects, antagonizing free radical production, and reducing expression of nitric oxide synthase and cyclooxygenase (55). In addition, Moradi et al. suggest that the PDE4 inhibitor roflumilast increases the polarization of macrophages toward the anti-inflammatory M2 phenotype, resulting in increased IL-10 and decreased TNF-α production (56).

Neuroprotective effects have also been demonstrated by Pearse et al., in rolipram increasing oligodendrocyte survival in an acute SCI model (46). Similarly, Beaumont et al. have shown that rolipram significantly increases oligodendrocyte survival in the ventrolateral funiculus (VLF) of the spinal cord following acute SCI, with improved VLF conductivity and significantly fewer footfalls in grid walk testing (44).

Additional mechanistic insights are available from studies of combination therapies. Iannotti et al. demonstrated that administering rolipram with clodronate significantly increased axonal sparing and BBB locomotor scores (51). In addition, Koopmans et al. found that administering rolipram with thalidomide increased white matter sparing at the SCI lesion center and significantly increased BBB locomotor scores (48). Nikulina et al. demonstrated that the addition of rolipram to a post-SCI embryonic stem cell transplant improved axonal growth into the transplant post-SCI (54). A single study using PDE4-I found significantly higher BBB scores than in vehicle-treated animals, a finding comparable to that of studies using rolipram (55). Similarly, a single study using roflumilast found BBB scores to be significantly improved compared with vehicle-treated animals (56).

### Evaluation of current methodologies and future perspectives

Significant heterogeneity exists between included studies. As a result, analysis of numerical effect estimates beyond study characteristics was not possible, and this review represents a qualitative synthesis of the literature. We have identified three key aspects within the methodologies of included studies that differed substantially: (1) the model of SCI utilized, (2) the intervention itself, including the PDE inhibitors that were delivered and the dosing-regimen, and (3) the neurobehavioral outcomes used to assess the efficacy of PDE inhibition in traumatic and non-traumatic SCI models.

Over the last 25 years, animal SCI models have become increasingly diverse. A range of injury mechanisms are now utilized, such as spinal cord contusion, compression, and transection (74). Differences exist even within individual SCI models; for example, spinal cord contusion may be induced using various types of impactor (75–78). As a result, the specific pattern of SCI induced, and subsequent pathophysiology, differs between the various models of SCI. This may in part explain inconsistencies between the results of included studies (79). No single model can replicate SCI in humans (80); researchers must therefore select an SCI model most suited to their research question. Most included studies that provided a rationale for SCI model choice, however, lacked detailed reporting of how the chosen model was implemented. This leads to difficulties in the replication of results (46, 62). A recent systematic review evaluating animal SCI models in the field of biomaterials similarly identified that poor reporting of methods and results had negatively impacted reproducibility in later studies (81). Comprehensive reporting of methodology in future studies would therefore aid result replication.

Significant variation between studies was also seen in the type of PDE inhibitor chosen and the mechanism of delivery. Currently, the relative merits of PDE inhibitors rolipram and cilostazol in the SCI context cannot be directly compared, as there is no overlap in the injury model or outcome measures for these two drugs in studies to date. Furthermore, in studies using the same PDE inhibitor, direct comparisons are hindered by variation in the route of administration and dosing regimens. It is also important to recognize the impact of differences in age, species and strain of animals. Not only would these factors have a significant impact on the pharmacodynamics of PDE inhibitors (82, 83), but they would also impact the pharmacokinetics, with differences in, for example, capillary permeability and local blood flow affecting drug absorption (84).

To aid clinical translation, it is important that routes of administration and dosing regimens amenable to the management of SCI in humans are considered when devising future experimental protocols. PDE inhibitor pharmacokinetic properties, including absorption, distribution, excretion, and metabolism, should also assessed (85). For example, rolipram readily crosses the blood–brain barrier (86), which is clinically advantageous in terms of being able to deliver the drug subcutaneously, whereas drugs that require a direct introduction to the site of injury may be less clinically translatable (54).

Finally, there was significant variation in the neurobehavioral outcomes used across included studies. Similar to models of SCI, each neurobehavioral outcome has its advantages and limitations; no single measurement can wholly assess the efficacy of PDE inhibitors in the context of SCI. For example, while the BBB score provides a simple and popular method to measure locomotion (87), identification of more subtle changes in motor recovery necessitates more intricate measures, such as 3D hindlimb kinematics (45). The most rigorous studies used a combination of neurobehavioral outcomes. Importantly, while our review focuses on neurobehavioral outcomes as a measure of PDE inhibitor efficacy, this is just one measure of efficacy. Other measures include immunohistochemistry, imaging, and neurophysiological parameters (79). While assessment of other forms of outcomes was beyond the scope of this review, it is important to acknowledge that the most robust studies assessed the efficacy of PDE inhibitors across multiple different outcome domains.

### Strengths and limitations

This review is the first to synthesize the impact of phosphodiesterase inhibition on neurobehavioral outcomes in preclinical models of traumatic and non-traumatic SCI. The review involved an exhaustive systematic literature search, a robust methodology that adheres to PRISMA guidelines and includes a robust risk of bias assessment using the SYRCLE tool.

Despite significant results in a majority of included studies, there was significant inconsistency in findings between studies. This may be explained by the diversity of interventions, with a range of injury techniques and dosing parameters used. Moreover, comparison between studies is limited by a lack of uniformity in the domains, methods, and timings of neurobehavioral assessment. In addition, while a majority of included studies reported positive results this may well not reflect a majority of studies conducted due to underreporting of negative results.

This review has utility in raising awareness of this heterogeneity; standardization of laboratory protocols used in future studies will improve interpretability and aid future synthesis. In addition, this study provides the fundamental preclinical background to clinical trials of phosphodiesterase inhibition in spinal cord injury, including the RECEDE-Myelopathy trial, which is currently evaluating the PDE4 inhibitor ibudilast in patients with degenerative cervical myelopathy (39).

## Conclusion

In preclinical models of traumatic and non-traumatic SCI, the exclusive administration of PDE inhibitors such as rolipram and cilostazol appeared to be associated with statistically significant improvements in neurobehavioral outcomes in a majority of included studies. However, evidence was inconsistent with a high risk of bias. Therefore, further evaluation of PDE inhibitors is required in the context of spinal cord injury to establish evidence of a repeatable and meaningful effect.

## Data availability statement

The original contributions presented in the study are included in the article/Supplementary material, further inquiries can be directed to the corresponding author.

## Author contributions

MB, OM, and BD: conceptualization, design, search strategy, and data interpretation. MB, OM, AB, UW, SR, TO, BW, LO, GS, IK, SV, RD, FB, F-E-MS, ARF, JT, BG, MA, and SA: screening. MB: data extraction and manuscript drafting. All authors: manuscript review. All authors contributed to the article and approved the submitted version.
