# Supplementary material for: The impact of phosphodiesterase inhibition on neurobehavioral outcomes in preclinical models of traumatic and non-traumatic spinal cord injury: a systematic review
Source: Front Med (Lausanne). 2023 Aug 22;10:1237219. doi: 10.3389/fmed.2023.1237219 (PMC10479944; doi:10.3389/fmed.2023.1237219)
Supplement: Supplementary file 1 [file Data_Sheet_1.PDF]

Supplementary material 1: Search Strategy and Results

The search was first performed on 11/09/19, then updated on 22/06/20 and 10/01/23. The result counts below are for 10/01/23.

#	Search terms	Results	
		Ovid MEDLINE(R) and Epub Ahead of Print, In-Process & Other Non-Indexed Citations, Daily and Versions(R) 1946 to January 10, 2023	EMBASE 1974 to 2023 January 10
1	(PDE3 or PDE-3 or phosphodiesterase 3 or PDE4 or PDE-4 or phosphodiesterase 4).mp.	4929	6750
2	exp Brain Injuries/	80339	205782
3	exp Nervous System Diseases/	2812739	4019022
4	exp Nerve Degeneration/	25969	73467
5	exp Spinal Diseases/	138260	274284
6	neurotrauma*.mp.	2618	4243
7	neurodegenerat*.mp.	150767	198604
8	((neuro* or brain* or nerve*) adj4 (trauma* or demyelinat* or remyelinat*)).mp.	71276	115303
9	((neuro* or brain* or nerve*) adj4 injur*).mp.	188485	300407
10	concussion*.mp.	16331	19608
11	or/2-10	3051610	4291488
12	1 and 11	488	1369

Supplementary Material 2: Full text articles excluded

Author (year)	Reason for exclusion
Myers et al. (2019)	PDE4b gene deletion. No PDE inhibitor drugs administered.
Boomkamp et al. (2014)	In vivo experiments only
Hinkle et al. (2005)	Sciatic nerve incision
Kajana et al. (2007)	No neurobehavioural outcomes
Macks et al. (2018)	No neurobehavioural outcomes
Whitaker et al. (2008)	No neurobehavioural outcomes
Liao et al. (2010)	Conference electronic poster
Puzis et al. (2010)	Conference abstract

Supplementary material 3: Risk of bias assessment

The Systematic Review Center for Laboratory Animal Experimentation (SYRCLE) Tool[28] was used to evaluate risk of bias.
Abbreviations: NS = not stated

**Bias assessment question**	Bao et al (2011)	Beaumont et al (2009)	Costa et al (2013)	Flora et al (2013)	Grosso et al (2013)	Iannotti et al (2011)	Nazli et al (2015)	Nikulina et al (2004)	Sahin et al (2011)	Schaal et al (2012)	Yamamoto et al (2014)
Was the allocation sequence adequately generated and applied?	Yes	Yes	Yes	Yes	Yes	Yes	Yes	NS	Yes	NS	NS
Were the groups similar at baseline or were they adjusted for confounders in the analysis?	Yes	Yes	Yes	NS	Yes	Yes	Yes	NS	NS	NS	Yes
Was the allocation adequately concealed?	NS	NS	NS	NS	NS	NS	NS	NS	NS	NS	NS
Were the animals randomly housed during the experiment?	NS	NS	NS	NS	NS	NS	NS	NS	NS	NS	NS
Were the caregivers and/or investigators blinded from knowledge which intervention each animal received during the experiment?	NS	NS	NS	NS	NS	NS	NS	NS	NS	NS	NS
Were animals selected at random for outcome assessment?	NS	NS	NS	NS	NS	NS	NS	NS	NS	NS	NS
Was the outcome assessor blinded?	Yes	Yes	Yes	Yes	Yes	Yes	Yes	Yes	Yes	Yes	NS
Were incomplete outcome data adequately addressed?	Yes	No<br>The data of 1 rat in the rolipram-treated group was removed from the study because of its morbidity.	Yes	No<br>21 of 90 removed from footprint and gridwalk tests due to inability to perform three consecutive steps. A disproportionate number were removed from	Yes	Yes	Yes	Yes	Yes	Yes	Yes

				groups not receiving rolipram.							
Are reports of the study free of selective outcome reporting?	Yes	Yes	Yes	Yes	Yes	Yes	Yes	Yes	Yes	Yes	Yes
Was the study apparently free of other problems that could result in high risk of bias?	Yes	Yes	Yes	Yes	Yes	Yes	Yes	Yes	Yes	Yes	Yes

**Bias assessment question**	Yin et al (2013	Bretzner et al (2010)	Pearse et al (2004)	Wang et al (2006)	Downing et al (2012)	Dai et al (2009)	Koopmans et al (2009)	Kurtoglu et al (2014)	Nout et al (2011	Sharp et al (2012)	Moradi et al (2020)
Was the allocation sequence adequately generated and applied?	Yes	NS	NS	NS	NS	NS	NS	Yes	NS	NS	Yes
Were the groups similar at baseline or were they adjusted for confounders in the analysis?	NS	Yes	NS	NS	NS	NS	NS	NS	Yes	NS	Yes
Was the allocation adequately concealed?	NS	NS	NS	NS	NS	NS	NS	NS	NS	NS	NS
Were the animals randomly housed during the experiment?	NS	NS	NS	NS	NS	Yes	NS	NS	NS	NS	NS
Were the caregivers and/or investigators blinded from knowledge which intervention each animal received during the experiment?	NS	NS	NS	NS	NS		NS	NS	NS	NS	NS
Were animals selected at random for outcome assessment?	NS	NS	NS	NS	NS	NS	NS	NS	NS	NS	NS
Was the outcome assessor blinded?	NS	Yes	NS	Yes	Yes	Yes	Yes	NS	NS	Yes	NS
Were incomplete outcome data adequately addressed?	Yes	Yes	Yes	Yes	No	Yes	No	Yes	No	No	Yes

					1 animal within the high-dose rolipram group was found deceased in its cage at week 6		6 of 74 animals died during the experiment (4 vehicle, 1 rolipram, 1 thalidomide)		5 of 45 animals died during the experiment (1 OP, 1 cAMP, 1 GRP, 2 GRP + cAMP)	Three rats that received surgery 8 days post-injury died due to surgical or anesthetic complications, and two others died the following day due to post-surgical complications. One rat from the Rolipram group was euthanized 7 days post- injury because the bladder ruptured during manual expression	
Are reports of the study free of selective outcome reporting?	Yes	Yes	Yes	Yes	Yes	Yes	Yes	Yes	Yes		Yes
Was the study apparently free of other problems that could result in high risk of bias?	Yes	Yes	Yes	Yes	Yes	Yes	Yes	Yes	Yes		Yes
